# Cholinergic enhancement reduces orientation-specific surround suppression but not visual crowding

**DOI:** 10.3389/fnbeh.2012.00061

**Published:** 2012-09-18

**Authors:** Anna A. Kosovicheva, Summer L. Sheremata, Ariel Rokem, Ayelet N. Landau, Michael A. Silver

**Affiliations:** ^1^Department of Psychology, University of California, BerkeleyBerkeley, CA, USA; ^2^School of Optometry, University of California, BerkeleyBerkeley, CA, USA; ^3^Helen Wills Neuroscience Institute, University of California, BerkeleyBerkeley, CA, USA; ^4^Department of Psychology, Stanford University, StanfordCA, USA; ^5^Ernst Strüngmann Institute for Neuroscience in Cooperation with Max Planck SocietyFrankfurt, Germany

**Keywords:** acetylcholine, surround suppression, crowding, pharmacology, psychophysics

## Abstract

Acetylcholine (ACh) reduces the spatial spread of excitatory fMRI responses in early visual cortex and receptive field size of V1 neurons. We investigated the perceptual consequences of these physiological effects of ACh with surround suppression and crowding, two phenomena that involve spatial interactions between visual field locations. Surround suppression refers to the reduction in perceived stimulus contrast by a high-contrast surround stimulus. For grating stimuli, surround suppression is selective for the relative orientations of the center and surround, suggesting that it results from inhibitory interactions in early visual cortex. Crowding refers to impaired identification of a peripheral stimulus in the presence of flankers and is thought to result from excessive integration of visual features. We increased synaptic ACh levels by administering the cholinesterase inhibitor donepezil to healthy human subjects in a placebo-controlled, double-blind design. In Experiment 1, we measured surround suppression of a central grating using a contrast discrimination task with three conditions: (1) surround grating with the same orientation as the center (parallel), (2) surround orthogonal to the center, or (3) no surround. Contrast discrimination thresholds were higher in the parallel than in the orthogonal condition, demonstrating orientation-specific surround suppression (OSSS). Cholinergic enhancement decreased thresholds only in the parallel condition, thereby reducing OSSS. In Experiment 2, subjects performed a crowding task in which they reported the identity of a peripheral letter flanked by letters on either side. We measured the critical spacing between the targets and flanking letters that allowed reliable identification. Cholinergic enhancement with donepezil had no effect on critical spacing. Our findings suggest that ACh reduces spatial interactions in tasks involving segmentation of visual field locations but that these effects may be limited to early visual cortical processing.

## Introduction

The neurotransmitter acetylcholine (ACh) plays an important role in cognitive functions such as attention, learning, and memory (Furey et al., 2000; Bentley et al., 2004; Sarter et al., 2005; Hasselmo, 2006; Kukolja et al., 2009; Rokem and Silver, 2010; Rokem et al., 2010; Newman et al., 2012). ACh also modulates the amplitude of responses to visual stimuli (Sato et al., 1987; Zinke et al., 2006) and stimulus selectivity (Sillito and Kemp, 1983; Sato et al., 1987; Murphy and Sillito, 1991; Zinke et al., 2006) of neurons in primary visual cortical area V1. An influential model of cholinergic effects on cortical processing proposes that ACh increases the efficacy of thalamocortical inputs relative to intracortical connections (Newman et al., 2012). In visual cortex, this mechanism would result in a decrease in the lateral spread of visual responses within the cortex. In addition, because the receptive fields of thalamocortical inputs are smaller than those of V1 cortical neurons (reviewed in Angelucci and Bressloff, 2006), a shift toward feedforward thalamocortical processing by ACh should reduce the excitatory receptive field size of V1 neurons.

These predictions have been supported by a number of animal neurophysiological and human brain imaging studies. ACh decreases the lateral spatial spread of excitation following electrical stimulation in rat visual cortical slices (Kimura et al., 1999) and reduces the stimulus length that evokes the maximal neuronal response of individual neurons in marmoset primary visual cortex (Roberts et al., 2005). In addition, administration of donepezil, a drug that increases synaptic levels of ACh, to healthy human subjects reduces the spatial spread of excitatory blood-oxygenation level dependent (BOLD) fMRI responses to visual stimuli in early visual cortex (Silver et al., 2008). Together, these results indicate that ACh plays a critical role in regulating spatial integration of visual responses across visual field locations.

These neurophysiological and human neuroimaging studies suggest that cholinergic transmission increases the spatial resolution of visual cortical stimulus representations. We hypothesized that this increased spatial resolution of cortical representations would have specific consequences for visual perception. In particular, cholinergic enhancement in human subjects should improve performance on tasks in which interactions between visual field locations normally impair performance. Specifically, we examined the effects of ACh in two types of tasks that require segmentation of visual field locations: (1) surround suppression of contrast discrimination, and (2) crowded letter identification.

Surround suppression typically refers to the reduction in neural responses (Hubel and Wiesel, 1968; Maffei and Fiorentini, 1976) and perceived contrast (Chubb et al., 1989; Cannon and Fullenkamp, 1993; Snowden and Hammett, 1998) of a target stimulus by simultaneous presentation of a high-contrast surround. Surround suppression is orientation-specific: it is stronger when the center and surround share the same orientation compared to when they are orthogonally oriented (Solomon et al., 1993; Xing and Heeger, 2001; Yoon et al., 2009, 2010). Primary visual cortex contains a large proportion of neurons that are selective for stimulus orientation (Hubel and Wiesel, 1959), and V1 neurons exhibit orientation-specific surround suppression (OSSS) of responses to visual stimuli presented to their classical receptive field (Blakemore and Tobin, 1972; Cavanaugh et al., 2002). In addition, the amount of surround suppression of fMRI visual responses to gratings in human V1 is highly correlated with behavioral measures of surround suppression (Zenger-Landolt and Heeger, 2003).

Another phenomenon related to perceptual spatial resolution is visual crowding, which refers to the impairment in the identification of a target peripheral stimulus in the presence of flanking stimuli. Increasing the distance between the targets and flanking stimuli improves the observer's ability to identify the target stimulus. Several studies have proposed that crowding results from excessive featural integration, in which pooling of features from adjacent crowded stimuli prevents the observer from individuating the stimuli (Chung et al., 2001; Levi et al., 2002b; Pelli et al., 2004). Other accounts attribute crowding to positional uncertainty or “source confusion” (Nandy and Tjan, 2007) or limitations on the resolution of visual spatial attention (He et al., 1996; Intriligator and Cavanagh, 2001). While many theories have been proposed to explain crowding (for reviews, see Levi, 2008; Whitney and Levi, 2011), little is known about its neural substrates.

The relationships between surround suppression and crowding are poorly understood. Both phenomena occur with peripheral stimuli and increase in magnitude as the stimulus eccentricity increases (Bouma, 1970; Toet and Levi, 1992; Petrov and McKee, 2006). Although both surround suppression and crowding are forms of inhibitory spatial interaction, it has been argued that surround suppression and crowding are behaviorally distinct (Levi et al., 2002b; Pelli et al., 2004; Petrov et al., 2007). Moreover, behavioral measurement of these two phenomena has typically involved different kinds of tasks: stimulus or target detection for surround suppression and stimulus identification for crowding.

Given the physiological evidence that cholinergic transmission increases spatial resolution of visual responses in early visual cortex (Kimura et al., 1999; Roberts et al., 2005; Silver et al., 2008), we examined the effects of ACh on visuospatial interactions in perception in two separate experiments. In each experiment, we increased synaptic levels of ACh in healthy human subjects by administering the Alzheimer's medication donepezil (Aricept®) using a placebo-controlled, double-blind procedure. Donepezil increases synaptic levels of ACh by inhibiting acetylcholinesterase, the enzyme responsible for breaking down ACh in the synaptic cleft.

In Experiment 1, subjects performed a contrast discrimination task within a central grating. Different experimental blocks contained one of the following: (1) a surrounding grating with the same orientation as the center (parallel surround, PS), (2) a surround perpendicular to the center (orthogonal surround, OS), or (3) no surround (NS). In Experiment 2, subjects reported the identity of a peripheral target letter flanked by a letter on either side. We quantified crowding by measuring critical spacing, defined as the target/flanker spatial separation required to achieve 80% accuracy (Pelli et al., 2007). We found that while cholinergic enhancement with donepezil decreased OSSS, it had no measurable effect on crowding. These results provide evidence for distinct neurochemical mechanisms underlying surround suppression and crowding.

## Materials and methods

### General methods

All subjects had normal or corrected-to-normal vision, provided informed consent, and were paid for their participation. Potential subjects who had a history of asthma or lung problems, neurological disorders, heart arrhythmia, or who were taking psychoactive medication or were regular cigarette smokers were excluded from participation. All testing was conducted in a dark soundproofed room with constant ambient light levels. Head position was stabilized with a chin rest. Stimuli were presented on a NEC Multisync FE992 CRT monitor. All experimental procedures were approved by the Committee for the Protection of Human Subjects at the University of California, Berkeley.

Donepezil and placebo were each administered in the form of a single capsule, prepared by the Drug Products Services Laboratory at the University of California, San Francisco. The dose of donepezil was 5 mg. For both experiments, a crossover design was used in which each subject received placebo before one session and donepezil before the other. Drug administration was double-blind, and the order of drug and placebo administration was counterbalanced between subjects. Testing started 3 h after the pill was administered, corresponding to the time of peak plasma concentration of donepezil after oral ingestion (Rogers and Friedhoff, 1998). At least 2 weeks passed between the drug/placebo sessions, allowing the drug, if present, to be completely eliminated (the half-life of donepezil is approximately 80 h; Rogers and Friedhoff, 1998).

### Experiment 1: surround suppression

#### Subjects

Nineteen subjects (10 female, mean age: 26 ± 2 years) participated in this experiment. Two subjects were excluded because their thresholds could not be reliably estimated due to high variability in the data. Specifically, the 95% confidence intervals for the contrast discrimination thresholds for these subjects, computed using a bootstrap procedure (Efron and Tibshirani, 1993), included values greater than 100% contrast. A third subject was excluded for failure to follow task instructions (this subject did not generate a response within the specified response window for a majority of the trials).

#### Visual stimuli and task

**Figure 1:**
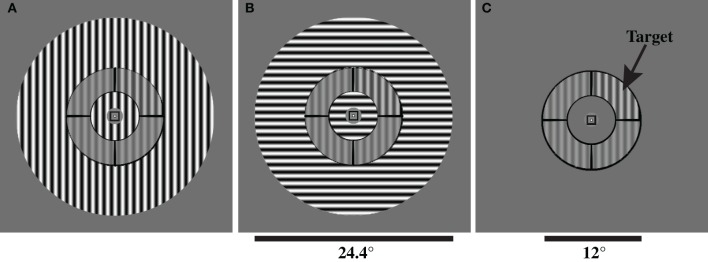
**Stimuli used to measure OSSS in Experiment 1.** Subjects performed a contrast discrimination task in one of three surround conditions: **(A)** parallel surround (PS), **(B)** orthogonal surround (OS), and **(C)** no surround (NS). Subjects reported which of the four segments of the annulus had higher contrast than the other three segments. The annulus and target are identical in all three panels.

The stimulus configuration and contrast discrimination task were adapted from Zenger-Landolt and Heeger (2003) and implemented with PsychoPy (Peirce, 2007). The stimulus was a circular patch containing a contrast-reversing (4 Hz), grayscale sinusoidal grating with a spatial frequency of 1.1 cycles per degree and was presented at a viewing distance of 60 cm. The stimulus was divided into annulus and surround regions by concentric black lines (Figure 1). The inner and outer radii of the annulus were 3° and 6° of visual angle, respectively. The surround region contained the remainder of the stimulus: the central portion, extending from the central fixation point to the inner border of the annulus, and the outer portion, extending from the outer border of the annulus to an eccentricity of 12.2° radius. Contrast was defined as: *C* = (*L*_max_ − *L*_min_)/(*L*_max_ + *L*_min_), where *L*_min_ and *L*_max_ were the minimal and maximal luminance values in a given stimulus, respectively. The surround contrast was always 0.8.

The annulus was divided into equal-sized quadrants, and the subject's task was to report which quadrant contained the contrast increment target. Each trial contained a contrast increment target that filled an entire quadrant of the annulus (equal probability of target presentation in each quadrant). Participants pressed one of four buttons to indicate the target location (4AFC), with each quadrant numbered between 1 and 4 in a clockwise rotation.

On each trial, the annulus and surround gratings were simultaneously presented for 750 ms, and the target contrast was linearly increased from the annulus (pedestal) contrast of 0.2 to a contrast determined by the psychophysical staircase (peak contrast occurred at the midpoint of the 750 ms stimulus presentation) and then linearly returned to a contrast of 0.2. The other three annulus segments contained uniform contrast of 0.2 throughout stimulus presentation.

The contrast difference between the pedestal and target segment was adjusted on each trial according to a 2-up, 1-down adaptive staircase procedure, converging to 71% accuracy (Levitt, 1971). A 2-up, 1-down staircase has previously been used to measure surround suppression of contrast detection (Zenger et al., 2000). Each staircase contained 80 trials, and the target contrast on the first trial of the staircase in each condition was determined based on the practice session before the first drug/placebo session. The use of an adaptive staircase ensured that all participants were performing the task near their psychophysical contrast discrimination thresholds. As a result, task difficulty (percentage of correct trials) was equated across all participants, for all three surround conditions (Figure 1), and for donepezil and placebo sessions.

The orientation of the grating within the annulus portion of the stimulus was either horizontal or vertical. Our inclusion of both horizontal and vertical annulus orientations was based on previous work demonstrating that both surround suppression (Kim et al., 2010) and orientation-specific overlay suppression (Essock et al., 2009) are greater for horizontal compared to vertically-oriented stimuli. In addition, the surround portion of the stimulus differed across three experimental conditions: (1) parallel surround (PS): surround and annulus shared the same orientation; (2) orthogonal surround (OS): surround and annulus orientations were perpendicular; (3) no surround (NS): stimulus contained only the annulus grating (Figure 1). The contrast reversals in the annulus (including the target) and surround gratings had the same temporal phase (i.e., contrast reversals were synchronous in the annulus and surround). In the PS condition, the annulus and surround gratings were collinear: they had the same spatial phase.

To compute the threshold in each condition, we plotted percent correct trials versus the difference between pedestal and target contrasts and then fit a Weibull cumulative distribution function to the data (Weibull, 1951). Thresholds were defined as the point of the Weibull function corresponding to 71% correct [the performance level to which the staircase converged (Levitt, 1971)]. The advantage to fitting the staircase data to a psychometric function is that the resulting threshold is based on all the trials in the staircase, in contrast to alternative methods of threshold estimation such as averaging reversals in the staircase or using only a small number of trials at the end of each run. Suppression indices were then quantified for each subject as ratios of these thresholds: PS/OS, PS/NS, and OS/NS. Variability in performance was assessed using a bootstrap procedure (Efron and Tibshirani, 1993) in which individual trials from a given condition and subject were resampled with replacement, and a psychometric curve was fit to this bootstrap sample. This procedure was iterated 10,000 times to produce a distribution of thresholds for each subject and surround condition. We defined performance variability as the 95th central percentile range of this distribution.

Statistical testing was performed using a mixed-model ANOVA, with drug condition (placebo vs. donepezil), annulus orientation (vertical vs. horizontal), and relative surround orientation (PS, OS, and NS) as within-subject factors. In order to account for potential training effects (e.g., better performance in later sessions due to practice), drug administration order (donepezil first vs. placebo first) was included in the ANOVA as a between-subjects factor. Mauchly's test of sphericity was performed when appropriate. In cases where the assumption of sphericity was not met, Greenhouse–Geisser corrections were applied.

#### Procedure

Each subject participated in three sessions. In the first session, a health screen was conducted, and informed consent was obtained. Following the screening procedure, eligible subjects were acquainted with the behavioral task and performed four psychophysical staircases (two vertical annulus, two horizontal annulus; 80 trials per staircase) for each surround condition (PS, OS, and NS). In each of the subsequent two sessions, subjects initially practiced an additional two staircases (one vertical annulus, one horizontal annulus) and then were administered a pill containing either donepezil or placebo. During each of the drug and placebo sessions, subjects performed four staircases (two horizontal annulus, two vertical annulus) for each surround condition (PS, OS, and NS; approximately 1 h of total testing).

### Experiment 2: crowding

Subjects performed two tasks in this experiment: (1) a letter acuity task, and (2) a crowded letter identification task. The letter acuity task was used to establish a letter size for each subject for the stimuli that were used in the crowding task. In order to ensure that limits on performance in the crowding task were not due to subjects' inability to resolve the letters, letter sizes were 1.5 times greater than each subject's letter acuity thresholds at each eccentricity.

#### Subjects

Eighteen subjects (12 females; mean age: 23 ± 6 years) participated in this experiment.

#### Visual stimuli and task

Stimuli were presented using a 2048 × 1536 screen resolution and a refresh rate of 60 Hz. Stimuli were generated using MATLAB (The MathWorks, Inc.) with the Psychophysics Toolbox (Brainard, 1997; Pelli, 1997). All stimuli were black on a white background and presented at maximal contrast. The viewing distance was 36 cm.

The fixation point was a square 0.1° on each side. Sloan letters were chosen for the letter acuity and crowding stimuli because all Sloan letters have a 1:1 height-to-width ratio and a stroke width equal to one-fifth the height of the letter. They are therefore commonly used for visual acuity testing. Each letter was selected randomly from the set of 10 standard Sloan letters (C, D, H, K, N, O, R, S, V, and Z), and the stimuli were centered at 5, 10, or 15 degrees eccentricity.

**Figure 2:**
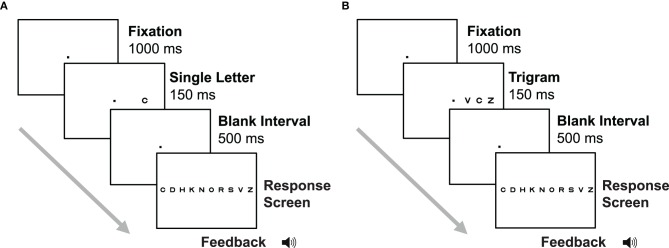
**Trial sequence for the letter acuity (A) and crowding (B) tasks in Experiment 2.** In the letter acuity task, subjects were presented with a single letter from a set of 10, in either the left or the right visual field. Subjects then reported the letter they saw by clicking on it on the response screen. In the crowding task, subjects were presented with three letters and reported the identity of the middle letter.

The trial sequence for the letter acuity task is shown in Figure 2. Each trial began with presentation of the fixation point at the center of the display for 1000 ms, followed by presentation of a single letter in either the left or right visual field (randomly selected) for 150 ms. This brief stimulus presentation was used to discourage eye movements, as saccades to the onset of a stimulus when the location is not known in advance typically take 200 ms to execute (Carpenter, 1988). After the letter presentation, there was a second fixation interval of 500 ms, followed by a response screen consisting of the full set of 10 Sloan letters arranged in a horizontal line. Letters on the response screen were 0.8° across and had a center-to-center separation of 4.5°. Subjects indicated which letter they saw by making a mouse click on that letter on the response screen. Auditory feedback was given on each trial. A high-pitched beep indicated a correct response, and a low-pitched beep was presented after incorrect responses. The crowding stimuli and trial sequence (Figure 2) were identical to the letter acuity stimuli, except that two flanking letters (each randomly selected from the set of 10 Sloan letters) were presented on either side of the target letter.

#### Procedure

***Training session.*** In the training session, initial estimates of letter acuity and critical spacing thresholds were determined using the method of constant stimuli. The training session consisted of three sets of trials, one for each eccentricity. Within each set, subjects performed the letter acuity and crowding tasks in alternating blocks of 70 trials each (total of four blocks). On each letter acuity trial, a letter size was randomly selected from a set of five equally spaced sizes. The ranges of letter sizes were 0.17–0.28°, 0.28–0.46°, and 0.4–0.9° in the 5, 10, and 15° eccentricity conditions, respectively. A similar procedure was used for the crowding task, in which a center-to-center spacing between the target letter and each flanker was selected from a set of five values. The ranges of target/flanker spacing were 2–3.4°, 3.8–6.7°, and 6.5–10°, and the letter sizes were 0.38, 0.56, and 0.98°, for the 5, 10, and 15° eccentricity conditions, respectively. The order in which subjects completed the three eccentricity sets was counterbalanced across subjects.

Letter acuity and critical spacing thresholds were calculated by fitting a Weibull function (Weibull, 1951) to the percent correct versus letter size or spacing data and then determining the letter size or spacing corresponding to 80% correct performance. The critical spacing of visual crowding has previously been defined as the target/flanker separation at which subjects achieve 80% correct target identification (e.g., Pelli et al., 2007). Thresholds were estimated separately for stimuli in the left and right visual field and then averaged for each subject and eccentricity.

During the training sessions, subjects' eye movements were manually monitored with an infrared camera during the first 10–15 min of the session. Subjects received feedback (in the form of a loud buzzing sound) when they made an eye movement away from central fixation. Trials in which eye movements occurred were excluded from subsequent analyses.

***Practice sessions.*** Subjects completed a short pre-test practice session the day before each of the two placebo/donepezil sessions. Practice sessions consisted of three sets, one for each eccentricity. Each set contained one block of 80 letter acuity trials and one block of 80 crowding trials. Letter size and spacing were adaptively varied according to a QUEST staircase procedure (Watson and Pelli, 1983), modified so that the stimulus level on each trial (as well as the final threshold estimate) was calculated based on the mean of the posterior probability density function of the subject's threshold (King-Smith et al., 1994). The staircase was set to converge to 80% accuracy for both tasks. The values of the slope parameter of the staircases were set to 3.7 for the letter acuity task and 1.6 for the crowding task, based on pilot measurements.

The stimulus level for the first trial of the staircase in each task in the practice session was equal to the subject's threshold from the training session. For the crowding task, letter size was equal to 1.5 times each subject's threshold in the letter acuity task, based on the training session data. Separate staircases of 40 trials each were collected in parallel for the left and right visual field (hemifield was randomly selected for each trial), and thresholds were calculated by averaging left and right visual field estimates.

***Test sessions.*** The procedure for the test sessions was identical to that of the practice sessions, except for the addition of two blocks of trials (80 letter acuity and 80 crowding trials) for each of the three eccentricities. Thus, for each eccentricity, there were four blocks of trials, alternating between the letter acuity task and the crowding task. The stimulus level for the first trial of the staircase in each task was equal to the subject's threshold from the first pre-test practice session. The letter size for the crowding task was equal to 1.5 times each subject's threshold in the letter acuity task, based on the first practice session.

## Results

### Experiment 1: surround suppression

In Experiment 1, we measured the effects of cholinergic enhancement with donepezil on contrast discrimination thresholds for three surround conditions: PS, OS, and NS (Figure 1). We employed a psychophysical staircase to calculate individual subject's thresholds in each condition. The use of a staircase procedure also served to equate task difficulty (% correct trials) and attentional demands across the different stimulus and drug conditions. We analyzed subjects' thresholds with a mixed-model ANOVA. The three within-subject factors were drug condition (placebo vs. donepezil), annulus orientation (vertical vs. horizontal), and relative surround orientation (PS, OS, and NS). In addition, we included drug administration order (donepezil first vs. placebo first) as a between-subjects factor.

**Figure 3:**
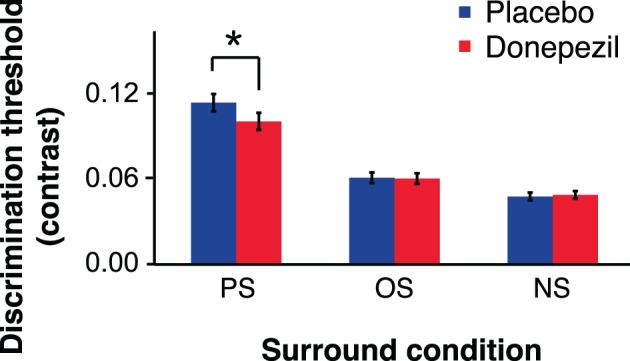
Contrast discrimination thresholds for parallel surround (PS), orthogonal surround (OS), and no surround (NS) conditions (^*^, *p* < 0.05). Error bars represent ±1 SEM of the within-subject difference between the placebo and donepezil conditions in each surround condition.

There was a significant main effect of relative surround orientation on contrast discrimination threshold [*F*_(1.14,15.96)_ = 31.28, *p* < 0.05] (Figure 3). There was no significant effect of annulus orientation (vertical vs. horizontal) and no significant interaction between annulus orientation and any other factor (all *p*-values >0.20). We therefore collapsed the data across the two annulus orientations and then recomputed thresholds for each relative surround orientation for each subject. To characterize the main effect of relative surround orientation in more detail, we performed paired *t*-tests comparing thresholds for the relative surround orientations. Thresholds for both PS and OS conditions were significantly higher than NS thresholds, for both placebo (PS vs. NS, *t*_(15)_ = 6.01, *p* < 0.05; OS vs. NS, *t*_(15)_ = 3.42, *p* < 0.05) and donepezil (PS vs. NS, *t*_(15)_ = 5.61, *p* < 0.05; OS vs. NS, *t*_(15)_ = 5.74, *p* < 0.05) sessions, demonstrating surround suppression. Furthermore, contrast thresholds were higher for the PS than the OS condition for both placebo (PS vs. OS, *t*_(15)_ = 5.63, *p* < 0.05) and donepezil (PS vs. OS, *t*_(15)_ = 4.43, *p* < 0.05) sessions, indicating that a component of this surround suppression was selective for the relative orientations of the annulus and surround.

In addition, the ANOVA revealed a significant interaction between relative surround orientation and drug condition (donepezil vs. placebo; *F*_(1.46, 20.44)_ = 5.34, *p* < 0.05). Paired *t-tests* showed that this interaction was due to a 12% decrease in PS thresholds following donepezil administration, relative to PS thresholds under placebo [*t*_(15)_ = 2.20, *p* < 0.05]. In contrast, there was no significant effect of donepezil on either NS [*t*_(15)_ = 0.045, *p* = 0.66] or OS [*t*_(15)_ = 0.18, *p* = 0.86] thresholds. Taken together, these results demonstrate that donepezil reduces OSSS by enhancing performance in the PS condition.

**Figure 4:**
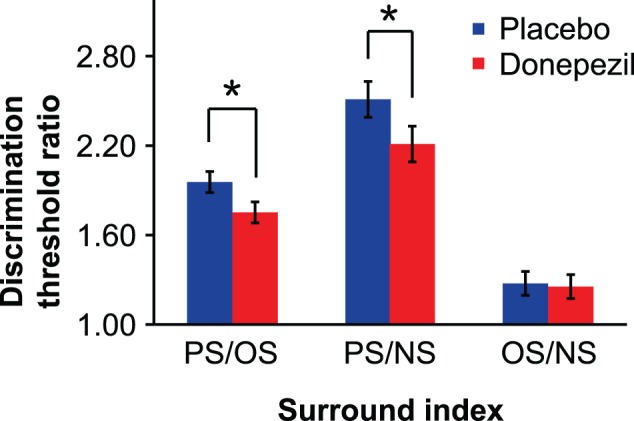
**Surround suppression indices.** Each index represents the ratio of contrast discrimination thresholds for parallel and orthogonal (PS/OS), parallel and no surround (PS/NS), or orthogonal and no surround (OS/NS) conditions. (^*^, *p* < 0.05). Error bars represent ±1 SEM of the within-subject difference between the placebo and donepezil conditions for each surround suppression index.

We computed ratios of contrast discrimination thresholds (PS/NS and OS/NS) in order to quantify suppression while controlling for variability in overall performance across subjects and sessions (e.g., due to non-specific drug effects on task performance or practice effects across sessions). Relative to placebo, donepezil administration significantly reduced PS/NS [*t*_(15)_ = 2.40, *p* < 0.05] but not OS/NS [*t*_(15)_ = 0.27, *p* = 0.79] ratios (Figure 4), providing further evidence that donepezil reduces OSSS. We tested this directly by computing the ratio of PS and OS thresholds for each session and found that donepezil significantly decreased PS/OS [*t*_(15)_ = 2.95, *p* < 0.05].

We did not observe a significant main effect of drug condition on overall contrast discrimination thresholds [*F*_(1,14)_ = 3.24, *p* = 0.093], suggesting that donepezil does not have a global effect on task performance. In addition, there was no main effect of drug administration order on thresholds [*F*_(1,14)_ = 1.81, *p* = 0.20], indicating that there was no difference in overall performance between the group of subjects that took placebo in their first session and the group that took donepezil first. However, there was a significant interaction between drug condition and drug administration order [*F*_(1,14)_ = 31.67, *p* < 0.05]. Because each subject had only one order of administration (either placebo then donepezil or donepezil then placebo), this interaction is due to differences in thresholds between the first and second sessions. Specifically, across surround conditions, subjects receiving placebo in their first session had lower overall thresholds on their subsequent donepezil session [*t*_(23)_ = 5.02, *p* < 0.05]. That is, there was an improvement in performance in the second (donepezil) session compared to the first (placebo) session. However, we did not observe this improvement across sessions in the group of subjects that received donepezil in their first session [*t*_(23)_ = 1.38, *p* = 0.18]. Importantly, our experimental design ensures that any improvement in performance in the second session relative to the first session is orthogonal to the effects of donepezil on surround suppression.

Finally, across participants, we did not observe a significant correlation between baseline values of the PS/OS ratio (obtained during the training sessions) and the magnitude of the reduction of this ratio by donepezil (*r* = −0.06, *p* = 0.82). This indicates that the initial strength of a subject's OSSS does not predict the size of the effect of cholinergic enhancement on OSSS.

### Experiment 2: crowding

In Experiment 2, we measured effects of cholinergic enhancement with donepezil on visual crowding. In separate blocks, subjects performed a letter acuity or visual crowding task.

#### Letter acuity thresholds

Subjects were presented with a single Sloan letter, either to the left or right of fixation, and then selected the letter that they perceived from a set of 10 possibilities (Figure 2A). Trials were blocked by eccentricity (5, 10, or 15 degrees), and letter size acuity thresholds were computed for each eccentricity. We analyzed letter acuity thresholds using a mixed-model ANOVA with within-subject factors of eccentricity (5, 10, or 15 degrees) and drug condition (donepezil or placebo). In addition, drug administration order (donepezil in first session vs. placebo in first session) was included as a between-subjects factor to account for potential training effects across sessions.

**Figure 5:**
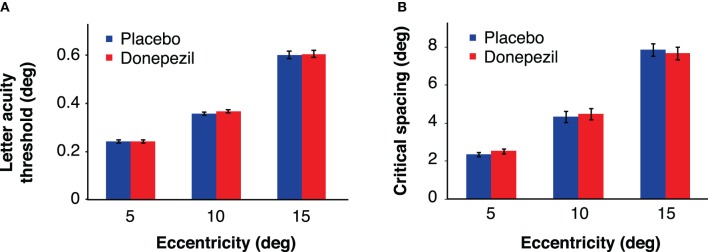
**Letter acuity thresholds (A) and critical spacing thresholds (B), defined as the letter size (acuity task) or the critical spacing (crowding task) corresponding to 80% correct performance.** Donepezil had no significant effect on either letter acuity or critical spacing at any eccentricity. Error bars represent ±1 SEM of the within-subject difference between the placebo and donepezil conditions at each eccentricity.

There was no significant main effect of drug [*F*_(1,16)_ = 0.77, *p* = 0.39] or drug administration order [*F*_(1,16)_ = 0.23, *p* = 0.64], indicating that neither donepezil nor the order of drug/placebo administration had an overall effect on letter acuity thresholds across sessions. The interaction between drug condition and drug administration order was also not significant [*F*_(1,16)_ = 0.62, *p* = 0.44]. However, we obtained the expected main effect of eccentricity [*F*_(2,32)_ = 287.65, *p* < 0.001], with larger letter acuity thresholds at more peripheral locations (Figure 5A). There were no significant interactions of eccentricity with any other factor (all *p*-values >0.13).

#### Critical spacing thresholds

In the crowding task, critical spacing was defined as the center-to-center spacing between the target letter and the flankers that resulted in 80% correct letter identification (Pelli et al., 2007). An ANOVA of these critical spacing thresholds revealed no overall effect of drug [*F*_(1,16)_ = 0.09, *p* = 0.77] or drug administration order [*F*_(1,16)_ = 3.07, *p* = 0.10]. As with the letter acuity thresholds, critical spacing increased with eccentricity [*F*_(2,32)_ = 146.37, *p* < 0.001] (Figure 5B). Critical spacing values were approximately equal to half of the eccentricity of the target letter, consistent with previous findings (Bouma, 1970).

There was also a significant interaction between drug condition and drug administration order across eccentricities [*F*_(1,16)_ = 7.12, *p* = 0.02]. Specifically, for subjects receiving donepezil in their first session, critical spacing thresholds were higher in that session than in the second placebo session [*t*_(26)_ = 2.10, *p* = 0.05]. There was no significant difference in critical spacing thresholds between the two sessions for subjects that received placebo in their first session [*t*_(26)_ = 1.35, *p* = 0.19]. This interaction between drug condition and drug administration order reflects an overall improvement in subjects' performance in the crowding task between the first and second sessions. A comparison of each subject's threshold in the first and second sessions (collapsing across the three eccentricity conditions) confirmed that critical spacing thresholds were lower in the second session compared to the first session [*t*_(17)_ = 2.74, *p* = 0.01], consistent with previous reports of learning effects in crowding over multiple training days (Chung, 2007; Hussain et al., 2012).

The interaction between eccentricity and drug administration order was also significant [*F*_(2,32)_ = 3.88, *p* = 0.03]. Specifically, in the 15° condition, subjects who received donepezil first had higher critical spacing thresholds (averaged across both sessions) than subjects who received placebo first [*t*_(16)_ = 2.26, *p* = 0.04]. There was no difference between placebo-first and donepezil-first groups in either the 5 degree (*p* = 0.58) or 10 degree (*p* = 0.40) conditions. Any significant effect of drug administration order suggests a possible influence of ACh on learning, because overall performance across the two sessions was influenced by which pill the subject received first (placebo or donepezil). However, our finding of worse performance for the donepezil-first group at 15 degrees eccentricity is inconsistent with a beneficial effect of cholinergic enhancement on learning in the crowding task. The drug condition × eccentricity interaction was not significant [*F*_(2,32)_ = 0.62, *p* = 0.55]. Finally, the three-way drug condition × drug administration order × eccentricity interaction was trending but not significant [*F*_(2,32)_ = 3.25, *p* = 0.052].

The correlation between subjects' critical spacing thresholds from the training session and the magnitude of the drug effect (donepezil threshold − placebo threshold) was not significant at any eccentricity (5 degrees: *r* = 0.07, *p* = 0.78; 10 degrees: *r* = −0.27, *p* = 0.27; 15 degrees: *r* = 0.17, *p* = 0.49) This indicates that, across participants, there was no relationship between the strength of crowding in the training session and the size of the drug effect on critical spacing.

#### Flanker substitution errors

**Figure 6:**
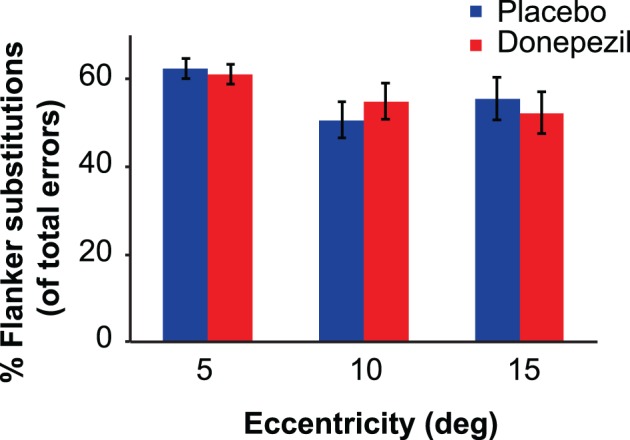
**Flanker substitution errors (expressed as percentage of total errors) in the crowding task.** Flanker substitution errors occur when the subject reports the identity of one of the two flanker letters instead of the target letter. Donepezil had no significant effect on the probability of flanker substitution errors at any eccentricity. Error bars represent ±1 SEM of the within-subject difference between the placebo and donepezil conditions at each eccentricity.

Finally, we examined the possibility that donepezil influenced the types of incorrect responses in the crowding task. One characteristic property of crowding in letter tasks is flanker substitution errors, in which subjects report one of the flanking letters to be the target letter (e.g., Strasburger, 2005). Because our staircase procedure adjusted critical spacing on each trial to maintain 20% incorrect responses for each subject, eccentricity, and session (donepezil or placebo), we analyzed the proportion of total errors that were flanker substitutions. On average, flanker substitution errors accounted for 56.2% of subjects' errors, far above the 11.1% value expected if incorrect responses were randomly distributed among the nine possibilities. Figure 6 shows the proportions of flanker substitution errors in donepezil and placebo conditions.

We applied the same mixed-model ANOVA to the proportions of flanker substitution errors that was used for the critical spacing thresholds. There was no significant main effect of drug condition [*F*_(1,16)_ = 0.002, *p* = 0.96] or drug administration order [*F*_(1,16)_ = 0.33, *p* = 0.57]. There was also no significant interaction between drug condition and drug administration order [*F*_(1,16)_ = 1.19, *p* = 0.29]. The main effect of eccentricity was significant [*F*_(1,16)_ = 7.41, *p* = 0.002]. Specifically, there was a significantly greater proportion of flanker errors at 5 degrees, compared to 10 degrees [*t*_(17)_ = 3.54, *p* = 0.003] and to 15 degrees [*t*_(17)_ = 3.15, *p* = 0.006]. There were no significant interactions between any other factors (all *p*-values > 0.22).

Together, the results from the crowding experiment indicate no significant effects of cholinergic enhancement on letter acuity, visual crowding, or the proportion of flanker substitution errors.

## Discussion

The results from Experiment 1 demonstrate that cholinergic enhancement in humans attenuates a perceptual measure of OSSS by improving performance in the PS condition. However, Experiment 2 shows that ACh does not affect all forms of spatial interactions in visual perception. In this experiment, we measured the minimum spacing between targets and flanking letters required to accurately identify the crowded target letter. We found that donepezil had no significant effect on critical spacing in visual crowding or on letter acuity. In addition, donepezil did not affect the proportion of flanker substitution errors.

The lack of a detectable effect of cholinergic enhancement on crowding in our experiment is unlikely to result from an inability to measure crowding. Subjects' ability to identify target letters 1.5 times larger than their acuity threshold was substantially impaired by flanking letters. Furthermore, the mean critical spacing at each eccentricity in our data has relatively low variance (Figure 5B), and the relationship between critical spacing and eccentricity is consistent with previous reports in the crowding literature (e.g., Bouma, 1970). Moreover, the inclusion of drug administration order as a between-subjects factor in the ANOVAs accounts for variance associated with possible training effects between sessions.

The effects of donepezil on OSSS and crowding were assessed at different threshold levels of performance (71% and 80%, respectively). To test if the differential effects of donepezil in the two experiments could have been related to differences in performance level, we reanalyzed the surround suppression data, measuring the contrast value on the Weibull function fits that corresponded to 80% performance. This was done for each combination of relative surround orientation and drug condition. As in our primary analysis at a 71% performance level, paired *t*-tests demonstrated a significant decrease in PS thresholds following donepezil administration, relative to placebo [*t*_(15)_ = 2.19, *p* < 0.05] but no significant effect of donepezil on either NS [*t*_(15)_ = 0.53, *p* = 0.61] or OS [*t*_(15)_ = 0.07, *p* = 0.94] thresholds. The effects of donepezil are therefore specific to the PS condition for both 71% and 80% performance levels, indicating that the absence of an effect of donepezil on crowding in Experiment 2 cannot be attributed to the fact that critical spacing was assessed at 80% performance. Furthermore, the percent correct values used to compare thresholds for donepezil and placebo conditions were identical within each experiment. This is also true for comparison of surround conditions in Experiment 1 and comparison of eccentricities in Experiment 2. In conclusion, the difference in percent correct values between the two experiments cannot account for our finding of a significant effect of donepezil on OSSS but not on crowding.

Our results demonstrate that ACh can have differential effects on contextual processing in visual perception. Crowding and surround suppression share a number of common features. For instance, both phenomena occur in the peripheral visual field and increase in magnitude with eccentricity (Bouma, 1970; Toet and Levi, 1992; Petrov and McKee, 2006). In addition, both crowding and surround suppression exhibit sensitivity to relative orientation (Solomon et al., 1993; Xing and Heeger, 2001; Levi et al., 2002a) and spatial frequency (Chubb et al., 1989; Chung et al., 2001).

Despite these similarities, there is also behavioral evidence to suggest that crowding and surround suppression are distinct phenomena. Unlike crowding (Levi et al., 2002b), surround suppression occurs in the fovea (Xing and Heeger, 2000). More broadly, it has been proposed that crowding differs from other forms of contextual interactions in that it affects identification rather than detection (e.g., Pelli et al., 2004). In surround suppression tasks, the target stimulus becomes more difficult to detect, presumably because of the decrease in perceived contrast. On the other hand, in crowding tasks, the stimulus remains clearly visible, but the observer cannot extract sufficient featural information to identify it. Finally, crowding is stronger when a single flanker is positioned on the peripheral side of the target compared to the foveal side (Bouma, 1970), but this anisotropy is not observed for surround suppression (Petrov et al., 2007).

While previous studies have documented behavioral differences between crowding and surround suppression, ours is the first to show that the two are dissociable using a pharmacological manipulation. One potential explanation for this dissociation is that crowding and surround suppression may occur at different stages of visual processing. Single unit studies have shown correlates of OSSS as early as the LGN (Sillito et al., 1993), and physiological correlates of surround suppression, as measured with electroencephalography and magnetoencephalography, have been localized to human primary visual cortex (Haynes et al., 2003). In addition, a behavioral measure of surround suppression in humans is better matched with fMRI correlates of surround suppression in V1 than in higher visual areas V2 and V3 (Zenger-Landolt and Heeger, 2003). The neural loci of crowding remain unknown, though there is behavioral (Louie et al., 2007; Liu et al., 2009) and fMRI (Bi et al., 2009; Freeman et al., 2011) evidence that crowding can occur at a later stage of visual cortical processing than area V1. It is possible, therefore, that the effects of ACh on OSSS result mainly from cholinergic modulation of early visual cortical representations.

The possibility of reduced OSSS by donepezil through an early visual cortical mechanism is consistent with reported physiological effects of ACh in early visual cortex. Specifically, ACh has been shown to reduce excitatory receptive field size in marmoset V1 (Roberts et al., 2005) and to decrease the spatial spread of the BOLD fMRI response to a stimulus in human early visual cortex (Silver et al., 2008). One possibility is that a cholinergic reduction in spatial integration in early visual cortical neurons specifically decreases the effects of the surround in the PS condition, resulting in less OSSS. These results support the hypothesis that ACh should improve performance in tasks in which spatial contextual interactions normally impair performance. On the other hand, the lack of an effect of donepezil on critical spacing in crowding may reflect a neural locus of crowding of letter stimuli that is later than early visual cortex in the visual processing hierarchy. Alternatively, neuronal receptive field size may not be the limiting factor in the excessive feature integration that characterizes crowding (e.g., Greenwood et al., 2010).

The differential effects of donepezil on OSSS and visual crowding may also be due to the well-documented effects of ACh on visual spatial attention (reviewed in Newman et al., 2012). Donepezil enhances the benefits of voluntary spatial attention on performance of a visual spatial cueing task (Rokem et al., 2010), and attentional modulation of V1 neuronal responses is blocked by local administration of the muscarinic ACh receptor antagonist scopolamine (Herrero et al., 2008). Spatial attention counteracts visual perceptual impairments due to surrounding distractors (Zenger et al., 2000; Roberts and Thiele, 2008), and directing attention to the center of a neuron's receptive field not only enhances responses to stimuli presented at the receptive field center but also reduces responses to visual stimuli presented to the unattended surround portion of the receptive field in macaque visual cortical areas MT (Anton-Erxleben et al., 2009) and V4 (Sundberg et al., 2009). Thus, spatial attention modulates perceptual and physiological measures of surround suppression. If cholinergic enhancement augments the effects of attention on surround suppression, this could account for our finding that donepezil reduces OSSS.

The effects of voluntary attention on crowding are much less clear. Crowding has been proposed to reflect the spatial resolution of voluntary attention (He et al., 1996; Intriligator and Cavanagh, 2001). However, to our knowledge, there have been no reports of modulation of critical spacing by voluntary attention, and crowding effects are robust even when voluntary spatial attention is fully allocated to the target location. If voluntary spatial attention influences OSSS more than crowding, cholinergic enhancement of attentional modulation would be expected to have greater effects on OSSS than on crowding, just as we have observed.

In conclusion, our results show that cholinergic enhancement in humans attenuates spatial interactions in OSSS but not in visual letter crowding. Our finding of dissociable neurochemical mechanisms underlying OSSS and crowding suggest that the effects of ACh may be limited to earlier stages of visual cortical processing.

### Conflict of interest statement

The authors declare that the research was conducted in the absence of any commercial or financial relationships that could be construed as a potential conflict of interest.
